# Radiation-induced severe lymphopenia predicts distant metastasis in rectal cancer: dosimetric implications for immune-sparing radiotherapy

**DOI:** 10.1186/s13014-026-02791-3

**Published:** 2026-01-29

**Authors:** Yi-Chiao Cheng, Wen-Yen Huang, Po-Chien Shen, Fu-Mei Chen, Meei-Shyuan Lee, Jen-Fu Yang, Hao-Cheng Chang, Wei-Chou Chang, Jia-Hong Chen, Cheng-Hsiang Lo

**Affiliations:** 1 Division of Colon and Rectal Surgery, Department of Surgery, Tri-Service General Hospital, National Defense Medical University, Taipei, Taiwan; 2 Department of Radiation Oncology, Tri-Service General Hospital, National Defense Medical University, No. 325, Sec 2, Cheng-Gong Rd, Neihu, Taipei, 114 Taiwan; 3 School of Public Health, National Defense Medical University, Taipei, Taiwan; 4 Department of Radiology, Tri-Service General Hospital, National Defense Medical University, Taipei, Taiwan; 5 Division of Hematology/Oncology, Department of Internal Medicine, Tri-Service General Hospital, National Defense Medical University, Taipei, Taiwan

**Keywords:** Rectal cancer, Radiation-induced lymphopenia, Distant metastasis, Pelvic bone marrow, Immune-sparing radiotherapy, Dosimetric analysis

## Abstract

**Background:**

Lymphopenia is a frequent complication of pelvic radiotherapy and may impair systemic immune surveillance. This study aimed to evaluate the prognostic impact of acute severe lymphopenia (ASL) in rectal cancer and to identify dosimetric predictors relevant to immune-sparing radiotherapy.

**Methods:**

Patients with non-metastatic rectal cancer treated with radiotherapy between 2018 and 2022 were retrospectively reviewed. Clinical variables, serum biomarkers (CEA and NLR), lymphocyte counts, and pelvic bone marrow dosimetry were analyzed. Associations between ASL, distant metastasis-free survival (DMFS), overall survival (OS), and dosimetric metrics were assessed using multivariable models.

**Results:**

A total of 161 patients were included, with 69.6% developing ASL. Patients with ASL had inferior 3-year DMFS (72.6% vs. 84.7%; *p* = 0.034), and ASL remained an independent predictor of poorer DMFS (*p* = 0.027). ECOG performance status ≥ 1 (*p* = 0.005), clinical N2 stage (*p* = 0.016), and baseline CEA > 5 ng/mL (*p* = 0.041) were also associated with worse DMFS, while adjuvant chemotherapy was protective (*p* < 0.001). Predictors of ASL included lower baseline absolute lymphocyte count (OR 0.87, 95% CI 0.81–0.93 per 0.1 × 10⁹/L increase; *p* < 0.001), higher clinical T stage (OR 2.38, 95% CI 1.01–5.56; *p* = 0.046), and greater low-dose irradiation to the lower pelvis—V5 (OR 1.06, 95% CI 1.02–1.11 per 1% increase; *p* = 0.005), for which the optimal predictive cut-off was 88%.

**Conclusion:**

ASL was associated with increased risk of distant metastasis. As baseline immunity and disease burden are non-modifiable, minimizing lower pelvic V5 using as low as reasonably achievable (ALARA)–based planning constraints may help reduce ASL risk and support immune-sparing radiotherapy in rectal cancer.

**Supplementary Information:**

The online version contains supplementary material available at 10.1186/s13014-026-02791-3.

## Background

Rectal cancer accounts for a substantial portion of the global colorectal cancer burden [1], with neoadjuvant concurrent chemoradiotherapy (CCRT) followed by surgery and adjuvant chemotherapy remaining the standard of care for locally advanced disease for decades [2, 3]. Although total neoadjuvant therapy has improved treatment compliance and systemic control [4–6], distant metastasis remains a major cause of treatment failure in over 20% of cases [5, 7].

Lymphocytes are central to antitumor immunity and host defense, but they are highly radiosensitive. Even low doses—such as 0.5 Gy—can induce substantial lymphocyte death [8], and the estimated lethal dose required to reduce the surviving fraction of circulating lymphocytes by 90% is as low as 3 Gy [9]. Radiotherapy depletes circulating lymphocytes and damages lymphopoietic organs such as bone marrow [10], leading to radiation-induced lymphopenia. This impairs systemic immune surveillance, increasing susceptibility to opportunistic infections [11] and the risk of cancer progression. Radiation-induced lymphopenia has been associated with inferior survival outcomes across various solid tumors, including lung, esophageal, and brain cancers [12–17].

However, in rectal cancer, the impact of pelvic bone marrow irradiation on lymphopenia—and its prognostic relevance, particularly for distant metastasis—remains underexplored. In this study, we investigated a cohort of patients with rectal cancer treated with pelvic radiotherapy. The objectives were to (1) evaluate the prognostic significance of acute severe lymphopenia (ASL) for survival outcomes and (2) identify clinical and dosimetric factors associated with ASL development, with a particular emphasis on practical parameters relevant to radiotherapy planning and the development of immune-sparing strategies.

## Methods

### Patient selection

We retrospectively reviewed medical records of all consecutive patients newly diagnosed with rectal cancer and treated with radiotherapy at our institution between January 2018 and December 2022. Inclusion criteria were: (1) histologically confirmed rectal adenocarcinoma, (2) curative-intent radiotherapy, and (3) available complete blood count (CBC) with differential at baseline and at least two additional CBCs at ≥ 1-week intervals throughout the course of radiotherapy. Patients were excluded if they had distant metastases at staging, synchronous malignancies, prior pelvic radiotherapy, or underwent upfront surgery followed by adjuvant radiotherapy.

This study was approved by the Institutional Review Board of the Tri-Service General Hospital (IRB No. B202505041), with a waiver of informed consent due to the study’s retrospective nature.

### Therapy and follow-up

All patients received long-course, conventionally fractionated pelvic radiotherapy (1.8–2 Gy per fraction). The standard prescribed radiation dose was 45 Gy in 25 fractions to the pelvis, with a local boost tailored individually based on dose constraints of organs at risk (OARs). Radiotherapy was administered using either three-dimensional conformal radiotherapy (3DCRT) or intensity-modulated radiation therapy (IMRT), including static beam, volumetric modulated arc therapy (VMAT), or helical tomotherapy (TOMO).

For 3DCRT, a standard four-field box technique was used based on classic anatomical landmarks, followed by opposed lateral boost fields [18]. For IMRT techniques, the clinical target volume of the pelvic field (CTV_pelvis_) encompassed the gross tumor and elective nodal regions, delineated according to the Radiation Therapy Oncology Group consensus atlas [19]. The boost CTV (CTV_boost_) comprised the gross tumor with a 2-cm longitudinal margin, incorporating the entire rectum, mesorectum, and presacral tissues. Planning target volumes (PTVs) were generated by expanding the CTVs by 5–8 mm. The bladder, small bowel, and bilateral femoral heads were designated as OARs. Radiation to the PTV_boost_ was delivered using either a simultaneous integrated or sequential boost technique, depending on institutional preference and dose constraints. Minor variations in target delineation were allowed based on individual patient anatomy and clinical judgment.

Concurrent fluorouracil-based chemotherapy was administered during radiotherapy when clinically appropriate. Concurrent chemotherapy regimens included 5-fluorouracil (5-FU), capecitabine, tegafur–uracil (UFT), oxaliplatin plus 5-fluorouracil/leucovorin (FOLFOX), or no concurrent chemotherapy. Post-treatment evaluation, including physical examination, imaging and laboratory testing, was typically performed within 1–2 months after radiotherapy. Surgery, when indicated, was performed 8–12 weeks after radiotherapy. Post-operative chemotherapy was recommended for patients with high-risk features, such as ypT3–T4 or node-positive disease, if tolerated. For patients managed non-operatively, consolidation chemotherapy was included as part of the treatment strategy, contingent on tolerance. Follow-up assessments were performed every 3 months afterward, as appropriate.

For analysis, chemotherapy administered after the completion of radiotherapy was categorized as adjuvant chemotherapy.

### Immune markers

Peripheral blood counts were typically evaluated at baseline, weekly during radiotherapy, at 1–2 months post-radiotherapy (commonly as part of the pre-surgical evaluation), prior to the initiation of adjuvant chemotherapy (if administered), and every 3 months thereafter for up to 1 year, as clinically appropriate. Total lymphocyte and neutrophil counts were derived from the CBC panel. The neutrophil-to-lymphocyte ratio (NLR) was calculated by dividing the peripheral neutrophil count by the lymphocyte count.

ASL was defined as a lymphocyte count of < 0.5 $\:\times\:$10^9^/L during or within 3 months after radiotherapy. The analysis of radiation-related ASL was censored at the time of subsequent surgery or adjuvant chemotherapy.

### Bone marrow delineation

The pelvic bone structures were not delineated as OARs during initial treatment planning. Pelvic bone marrow (PBM) was retrospectively contoured on the planning computed tomography images using bone window settings, with the contouring process blinded to patients’ CBC data. The PBM was divided into three subsites—lumbosacral spine (LS), iliac crests (ICs), and lower pelvis (LP), as described by Mell et al. [20]. To ensure consistency, all PBM subsites were contoured by a single staff member. Dose distributions for each bony subsite and the entire PBM were extracted from the initial treatment plan. Patients were included in the dosimetric analysis only if complete digital radiotherapy planning files were available to allow full extraction of PBM dose–volume metrics. Dose–volume data were reported as mean dose and volume percentages receiving more than x Gy (Vx). All treatment plans were reviewed using the Pinnacle planning system (version 16.2; Philips Radiation Oncology Systems, Fitchburg, WI, USA).

### Statistical analysis

All statistical analyses were performed using SPSS software (version 25, SPSS Inc., Chicago, IL, USA) and R software (version 4.2.0, R Foundation for Statistical Computing, Vienna, Austria). Continuous variables were compared using the Student’s t-test, whereas categorical variables were analyzed using the chi-square test. Survival outcomes, including distant metastasis-free survival (DMFS) and overall survival (OS), were estimated using the Kaplan–Meier method, with time measured from the initiation of radiotherapy (first day of treatment). DMFS was defined as the interval until documented distant metastasis or last follow-up. Patients who died without evidence of distant metastasis were censored at the time of death. OS was defined as the time to death from any cause or last follow-up. Intergroup comparisons of survival outcomes were conducted using the log-rank test. The Cox proportional hazards model was applied to assess associations between survival outcomes and relevant variables.

To address the potential impact of non-distant failure-related deaths on DMFS estimation, a competing risk analysis was performed using the Fine–Gray subdistribution hazard model. The cumulative incidence of distant metastasis was calculated, treating non-distant-failure deaths as competing events, and compared between groups using Gray’s test.

Concurrent chemotherapy regimen was included as a four-level categorical covariate in all regression models, with 5-FU or capecitabine—both recommended as standard CCRT regimens in international guidelines—as the reference category and UFT, FOLFOX, and no concurrent chemotherapy as comparator groups. NLR was modeled as a binary variable using a cut-off of 3, a threshold frequently adopted in rectal cancer CCRT studies [21]. This approach also minimized multicollinearity with baseline neutrophil and lymphocyte counts, which were included as continuous variables.

Because dosimetric data were not normally distributed, Spearman’s rank correlation coefficient (ρ) was used to assess correlations among dosimetric parameters, whereas the Mann–Whitney U-test was employed for intergroup comparisons of these parameters. Receiver operating characteristic (ROC) curve analysis was conducted to determine optimal cutoff values for predicting ASL, with the model’s discriminative performance evaluated using the area under the ROC curve (AUC). The dosimetric parameter identified through ROC analysis, along with its corresponding cutoff value, was incorporated into univariable and multivariable logistic regression analyses to evaluate associations between ASL occurrence and clinical or dosimetric variables.

For both Cox and logistic regression models, variables with p-values < 0.1 in univariable analyses were considered candidates for multivariable analyses. Given the limited number of outcome events, a backward stepwise selection procedure based on likelihood-ratio tests was applied to optimize model complexity and avoid overparameterization. Variance inflation factors (VIFs) were calculated for all variables retained in the final multivariable models; all VIF values were < 2, indicating no concerning multicollinearity. Two-tailed p-values < 0.05 were considered statistically significant for all analyses.

## Results

### Patient characteristics

Among 220 consecutive patients diagnosed with rectal cancer and treated with radiotherapy during the study period, 59 were excluded based on predefined criteria (Fig. S1, Supplementary material), leaving 161 patients for analysis (Table 1). In total, 155 patients (96.3%) were treated with CCRT, and six patients (3.7%) were treated with radiotherapy alone. The most common radiation dose was 50–50.4 Gy in 25–28 fractions (*n* = 140, 86.9%), followed by 54 Gy in 30 fractions (*n* = 10, 6.2%). Concurrent chemotherapy was most commonly UFT (33.5%) and 5-FU (31.1%), with FOLFOX administered in 29.2% of patients. Surgery and adjuvant chemotherapy were performed in 120 (74.5%) and 118 (73.3%) patients, respectively.

**Table 1:** Patient and treatment characteristics (*n* = 161)

Characteristics	
Age, median (25th–75th), y	65 (57–74)
Male	105 (65.2)
ECOG-PS	
0	98 (60.9)
1	54 (33.5)
2	7 (4.3)
3	2 (1.2)
Clinical T stage	
2	38 (23.6)
3	110 (68.3)
4	13 (8.1)
Clinical N stage	
0	54 (33.5)
1	76 (47.2)
2	31 (19.3)
Clinical stage,	
Ⅰ	20 (12.4)
Ⅱ	34 (21.1)
Ⅲ	107 (66.5)
Distance from anal verge on endoscopy, cm	
< 5	35 (21.7)
5–10	68 (42.2)
≥ 10	58 (36.0)
Baseline blood cell count, median (25th–75th)	
Leukocyte, × 10⁹/L	6.92 (5.48–8.49)
Neutrophil, × 10⁹/L	4.29 (3.32–5.61)
Lymphocyte, × 10⁹/L	1.66 (1.28–2.09)
NLR, median (25th–75th)	2.6 (2.0–3.4)
BMI, median (25th–75th), kg/m^2^	24.1 (21.9–25.8)
CEA, median (25th–75th), ng/mL	3.9 (2.4–10.3)
Concurrent chemotherapy	
None	6 (3.7)
5-FU	50 (31.1)
Capecitabine	4 (2.5)
UFT	54 (33.5)
FOLFOX	47 (29.2)
Adjuvant chemotherapy	118 (73.3)
RT dose	
45 Gy	4 (2.5)
50–50.4 Gy	140 (86.9)
54 Gy	10 (6.2)
55.8 Gy	1 (0.6)
59.4 Gy	6 (3.7)
RT fraction, median (min–max)	28 (25–33)
RT duration, median (25th–75th), day	41 (39–43)
RT technique	
3DCRT	2 (1.2)
Static IMRT	4 (2.5)
VMAT	135 (83.9)
TOMO	20 (12.4)
Surgery	120 (74.5)

Data are presented as no. (%) unless otherwise indicated

Abbreviations: 3DCRT = 3D conformal radiotherapy; BMI = body mass index; CEA = carcinoembryonic antigen; ECOG-PS = Eastern Cooperative Oncology Group performance status; FOLFOX = Oxaliplatin plus 5-fluorouracil/leucovorin; IMRT = intensity-modulated radiotherapy; NLR = neutrophil-to-lymphocyte ratio; RT = radiotherapy; UFT = tegafur-uracil; VMAT = volumetric modulated arc therapy; TOMO = Helical Tomotherapy

### ASL and effect on survival outcomes

The median lymphocyte count of all patients at baseline was 1.66 × 10^9^/L (25th–75th, 1.28–2.09). Overall, 112 (69.6%) patients experienced ASL, with a median date of development of 39 (25th–75th, 31–47) days after the beginning of radiotherapy; of these, two had baseline lymphocyte counts < 0.5 × 10^9^/L.

**Fig. 1:**
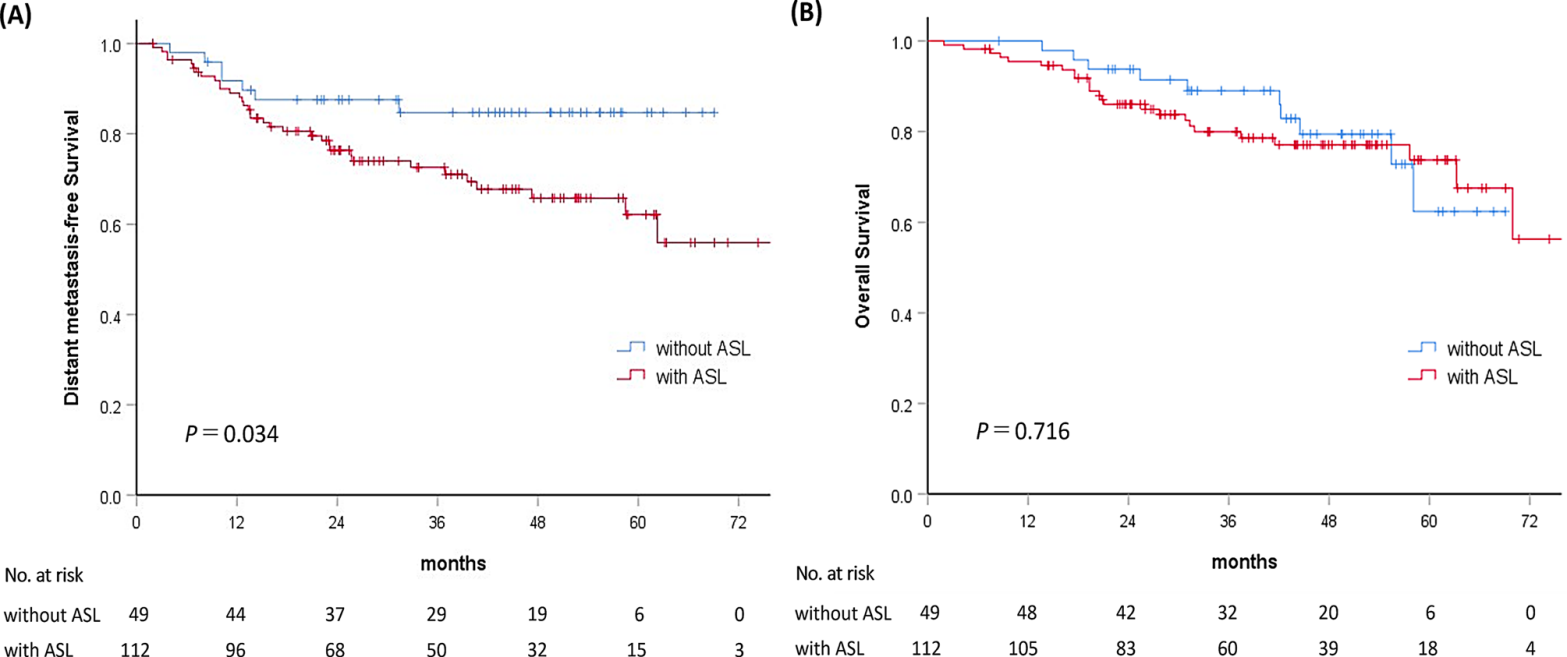
Kaplan–Meier curves of distant metastasis-free survival (**A**) and overall survival (**B**) stratified by the presence of ASL. Abbreviation: ASL = acute severe lymphopenia

The median follow-up duration was 41.5 (25th–75th, 24.5–53.7) and 44.9 (25th–75th, 28.8–55.0) months for all patients and those still alive (*n* = 126, 78.3%), respectively. During the follow-up period, 41 (25.5%) patients experienced distant metastasis and the 1- and 3-year DMFS rates were 89.9% and 76.3%, respectively. The 3-year DMFS for patients with ASL was 72.6% compared with 84.7% for patients without ASL (*p* = 0.034; Fig. 1A). In univariable Cox regression analysis, Eastern Cooperative Oncology Group performance status (ECOG-PS) ≥ 1, clinical N2 stage, NLR ≥ 3, lower baseline absolute lymphocyte count (ALC), baseline carcinoembryonic antigen (CEA) > 5 ng/mL, absence of adjuvant chemotherapy, and the presence of ASL were associated with poorer DMFS (all *p* < 0.1) (Table 2).

**Table 2:** Prognostic factors on DMFS and OS by Cox proportional-hazards model

	DMFS				OS			
	UVA		MVA		UVA		MVA	
	HR (95% CI)	*p*	HR (95% CI)	*p*	HR (95% CI)	*p*	HR (95% CI)	*p*
Age > 65 years	1.65 (0.89–3.08)	0.114			3.98 (1.85–8.56)	< 0.001	2.85 (1.19–6.83)	0.019
Female vs. male	0.99 (0.52–1.88)	0.968			1.44 (0.73–2.83)	0.295		
ECOG-PS ≥ 1 vs. 0	2.36 (1.27–4.38)	0.006	2.63 (1.33–5.18)	0.005	7.46 (3.25–17.12)	< 0.001	5.94 (2.41–14.64)	< 0.001
Clinical T stage								
T3–T4 vs. T2	1.33 (0.59–3.01)	0.488			0.93 (0.42–2.04)	0.848		
Clinical N stage								
N1 vs. N0	1.39 (0.65–2.97)	0.394	2.00 (0.91–4.41)	0.085	0.94 (0.44–2.02)	0.883		
N2 vs. N0	2.07 (0.88–4.87)	0.096	3.17 (1.24–8.13)	0.016	1.11 (0.43–2.88)	0.824		
Clinical stage Ⅲ vs. Ⅰ–Ⅱ	1.58 (0.77–3.21)	0.212			0.99 (0.48–2.02)	0.975		
Distance from anal verge, cm								
5–10 vs. <5 ≥ 10 vs. <5	1.42 (0.63–3.21) 0.79 (0.32–1.93)	0.401 0.602			1.33 (0.57–3.08) 0.60 (0.23–1.56)	0.510 0.291		
Baseline neutrophil count (per 0.1 × 10⁹/L increase)	1.00 (0.99–1.02)	0.539			0.99 (0.97–1.01)	0.291		
Baseline lymphocyte count (per 0.1 × 10⁹/L increase)	0.95 (0.90–1.01)	0.073			0.94 (0.88–0.99)	0.022		
NLR ≥ 3	1.76 (0.95–3.24)	0.072	1.75 (0.91–3.36)	0.096	1.40 (0.72–2.73)	0.329		
BMI (per 1 kg/m^2^ increase)	0.94 (0.85–1.03)	0.199			0.88 (0.79–0.98)	0.018	0.86 (0.78–0.96)	0.008
CEA > 5 ng/mL	2.04 (1.10–3.77)	0.024	1.99 (1.03–3.85)	0.041	1.18 (0.61–2.31)	0.621		
Concurrent chemotherapy^†^								
None vs. 5-FU/Capecitabine UFT vs. 5-FU/Capecitabine FOLFOX vs. 5-FU/Capecitabine	0.72 (0.09–5.50) 1.25 (0.58–2.71) 1.47 (0.69–3.14)	0.750 0.566 0.315			7.77 (2.08–29.06) 5.62 (2.09–15.10) 1.71 (0.54–5.41)	0.002 0.001 0.359	1.40 (0.32–6.10) 3.24 (1.17–9.01) 4.00 (1.21–13.23)	0.653 0.024 0.023
Adjuvant chemotherapy	0.39 (0.21–0.73)	0.003	0.23 (0.11–0.46)	< 0.001	0.25 (0.13–0.50)	< 0.001	0.24 (0.11–0.52)	< 0.001
RT dose > 50.4 Gy	1.36 (0.53–3.47)	0.524			2.03 (0.78–5.32)	0.148		
ASL	2.35 (1.04–5.31)	0.039	2.61 (1.12–6.11)	0.027	1.15 (0.55–2.40)	0.716		
Surgery	0.66 (0.34–1.30)	0.228			0.44 (0.22–0.87)	0.018		

^†^Reference group = 5-FU/Capecitabine

Abbreviations: ASL = acute severe lymphopenia; BMI = body mass index; CEA = carcinoembryonic antigen; CI = confidence interval; DMFS = distant metastasis-free survival; ECOG-PS = Eastern Cooperative Oncology Group performance status; FOLFOX = Oxaliplatin plus 5-fluorouracil/leucovorin; HR = hazard ratio; MVA = multivariable analysis; NLR = neutrophil-to-lymphocyte ratio; OS = overall survival; RT = radiotherapy; UFT = tegafur-uracil; UVA = univariable analysis

In the univariable Fine–Gray competing risk analysis, ASL was significantly associated with an increased risk of distant metastasis (subdistribution hazard ratio: 2.33; 95% confidence interval [CI]: 1.03–5.29; *p* = 0.043). The cumulative incidence function curves similarly showed a higher incidence of distant metastasis in the ASL group compared to the non-ASL group (Gray’s test, *p* = 0.034; Fig. S2, Supplementary material).

In multivariable Cox regression analysis, ASL (hazard ratio [HR], 2.61; 95% CI 1.12–6.11; *p* = 0.027), ECOG-PS ≥ 1 (HR 2.63; 95% CI 1.33–5.18; *p* = 0.005), clinical N2 stage (HR 3.17; 95% CI 1.24–8.13; *p* = 0.016), and baseline CEA > 5 ng/mL (HR 1.99; 95% CI 1.03–3.85; *p* = 0.041) were predictive of worse DMFS, while adjuvant chemotherapy remained protective (HR, 0.23; 95% CI 0.11–0.46; *p* < 0.001). No concerning multicollinearity was identified among variables included in the multivariable model. In the overall cohort, no significant ASL×T-stage interaction was observed for DMFS in the multivariable Cox model (*p* for interaction = 0.115), suggesting no strong evidence that the association between ASL and DMFS differs by clinical T stage (T2 vs. T3–4).

The 3-year OS was 79.9% versus 89.0% in the ASL and non-ASL groups respectively, with no significant difference (*p* = 0.716; Fig. 1B). ASL showed no association with OS in univariable analysis, and this remained nonsignificant after multivariable adjustment. Independent predictors of OS included age, ECOG-PS, body mass index (BMI), concurrent chemotherapy regimen, and receipt of adjuvant chemotherapy. Subgroup and interaction analyses were performed to further assess whether the limited impact of ASL on OS varied across clinical contexts. Among patients who did not receive adjuvant chemotherapy, ASL was not associated with OS (HR 1.83; 95% CI 0.68–4.90; *p* = 0.231). Similarly, in patients who received adjuvant chemotherapy, ASL showed no association with OS (HR 1.10; 95% CI 0.35–3.43; *p* = 0.873). Interaction terms were then evaluated to determine whether specific clinical factors modified the association between ASL and OS. No significant interaction was observed for age (*p* = 0.147) or ECOG-PS (*p* = 0.128). In contrast, a statistically significant interaction between ASL and BMI was identified (*p* = 0.031), indicating that the impact of ASL on OS varied by BMI. The negative interaction coefficient suggests that higher BMI attenuated the adverse effect of ASL on OS.

### Clinicopathological and dosimetric predictors of ASL

The relationships between ASL and clinicopathological features were detailed in Supplementary material (Table S1). A higher clinical T stage (*p* = 0.029), a lower baseline ALC (*p* < 0.001), and NLR ≥ 3 (*p* = 0.005) were significantly associated with ASL development. The incidence of ASL did not differ significantly among patients treated with 5-FU/capecitabine, UFT, FOLFOX, or no concurrent chemotherapy (overall *p* = 0.441). Among the 154 patients evaluable for dose–volume parameters, 105 developed ASL (Table 3). Seven patients (4.3%) were not evaluable because their digital radiotherapy planning files were partially damaged or incomplete, leaving only printed plan summaries without reconstructable dosimetric data. A comparison of the original cohort with the patients evaluable for dosimetry revealed no evidence of selection bias (Table S2, Supplementary material).

**Table 3:** Relationships between the development of ASL and dose volume parameters

Dose volume parameter	Cohort without ASL (*n* = 49)	Cohort with ASL (*n* = 105)	*p*
Iliac crests			
V5, %	98.0 (93.0–100.0)	99.0 (95.5–100.0)	0.310
V10, %	92.0 (87.0–96.5)	92.0 (87.5–98.0)	0.568
V15, %	83.0 (77.0–89.0)	84.0 (77.0–88.5)	0.698
V20, %	71.0 (64.0–76.0)	71.0 (66.0–77.0)	0.560
V30, %	42.0 (36.5–49.0)	44.0 (36.0–50.0)	0.524
V40, %	20.0 (15.5–24.5)	21.0 (15.5–27.0)	0.230
V50, %	2.0 (1.0–5.0)	3.0 (1.0–6.0)	0.164
Dmean, Gy	27.7 (26.0–29.9)	27.8 (26.2–30.6)	0.458
Lumbosacral spine			
V5, %	99.0 (92.0–100.0)	100.0 (94.5–100.0)	0.253
V10, %	94.0 (83.5–99.0)	97.0 (86.0–100.0)	0.132
V15, %	89.0 (80.0–97.0)	94.0 (82.0–99.5)	0.117
V20, %	86.0 (76.0–93.0)	91.0 (79.0–98.0)	0.096
V30, %	74.0 (67.5–82.5)	80.0 (69.0–86.5)	0.075
V40, %	58.0 (51.0–67.0)	61.0 (53.0–69.0)	0.112
V50, %	21.0 (13.0–33.0)	25.0 (18.0–32.0)	0.322
Dmean, Gy	38.1 (35.4–40.9)	39.8 (35.4–42.7)	0.077
Lower pelvis			
V5, %	94.0 (86.0–99.0)	97.0 (93.0–100.0)	**0.017**
V10, %	85.0 (71.0–92.0)	89.0 (82.0–94.0)	**0.044**
V15, %	71.0 (56.0–84.0)	74.0 (64.5–83.0)	0.098
V20, %	57.0 (41.5–67.5)	57.0 (48.0–68.0)	0.263
V30, %	29.0 (18.0–35.5)	28.0 (22.5–35.0)	0.701
V40, %	11.0 (6.5–14.0)	11.0 (8.0–15.0)	0.162
V50, %	1.0 (0.0–2.0)	1.0 (0.0–2.0)	0.834
Dmean, Gy	23.0 (19.0–26.0)	23.7 (20.9–26.1)	0.183
Pelvic bone marrow			
V5, %	95.0 (91.0–98.5)	97.0 (93.0–99.0)	**0.040**
V10, %	88.0 (82.0–94.0)	91.0 (85.5–94.0)	**0.041**
V15, %	78.0 (70.5–84.5)	81.0 (75.0–87.0)	0.066
V20, %	67.0 (60.0–74.5)	70.0 (63.0–76.5)	0.163
V30, %	45.0 (40.0–49.0)	46.0 (39.0–52.5)	0.361
V40, %	25.0 (21.0–29.0)	27.0 (21.5–32.5)	0.151
V50, %	6.0 (4.0–10.0)	8.0 (5.0–10.0)	0.325
Dmean, Gy	28.0 (25.7–29.7)	28.7 (26.4–31.8)	0.139

Data are presented as median (25th–75th)

Abbreviations: ASL = acute severe lymphopenia; Vx = volume of a structure receiving more than x Gy

Patients with ASL exhibited significantly higher V5 and V10 values for the LP and PBM, whereas dosimetric parameters for the LS and ICs did not significantly affect ASL development (Table 3). A strong correlation was observed between LP and PBM V5 and V10 values (ρ = 0.725, *p* < 0.001), and the LP accounted for a substantial proportion of the PBM volume (median: 41.9%; 25th–75th: 40.5–43.4%). This suggests that the statistical significance of the low-dose exposure to the whole PBM was primarily driven by the contribution of the LP.

ROC analysis demonstrated modestly better discriminative performance for LP V5 (AUC 0.62, 95% CI 0.52–0.72; *p* = 0.018) than for LP V10 (AUC 0.60, 95% CI 0.50–0.70; *p* = 0.044). These AUC values and the associated cut-off point were derived directly from ROC analyses performed in SPSS (no separate table). The optimal cut-off for LP V5 was 88%, yielding a sensitivity of 85.7% and a specificity of 38.8%. Among patients with LP V5 ≤ 88% (*n* = 34), the ASL rate was 44.1%, compared with 75.0% in those with LP V5 > 88% (*n* = 120) (*p* = 0.001). Given its modestly superior discrimination and greater biological plausibility in reflecting lymphocyte radiosensitivity at very low doses, LP V5 was therefore retained as the primary dosimetric parameter for inclusion in the final multivariable logistic regression model.

The choice of IMRT technique influenced low-dose distribution: TOMO plans were associated with significantly higher LP V5 (*p* = 0.007), whereas VMAT plans had slightly higher V50 values (*p* = 0.030). Full comparisons are provided in Supplementary material (Table S3).

Univariable analysis identified clinical T stage, baseline ALC, NLR ≥ 3, and the continuous LP V5 and V10 parameters as significantly associated with ASL development, whereas concurrent chemotherapy regimen was not significantly associated with ASL risk. In the multivariable model incorporating LP V5, LP V5 (OR, 1.06; 95% CI 1.02–1.11 per 1% increase; *p* = 0.005), lower baseline ALC (OR, 0.87; 95% CI 0.81–0.93 per 0.1 × 10⁹/L increase; *p* < 0.001), and higher clinical T stage (OR 2.38, 95% CI 1.01–5.56; *p* = 0.046) remained independent predictors of ASL development (Table 4). No concerning multicollinearity was observed, with all VIF values < 2 in the final model.

**Table 4:** Univariable and multivariable analyses of factors associated with ASL after radiotherapy

	UVA		MVA	
	OR (95% CI)	*p*	OR (95% CI)	*p*
Age > 65 years	1.32 (0.67–2.59)	0.422		
Female vs. male	1.73 (0.82–3.62)	0.148		
ECOG-PS				
≥ 1 vs. 0	1.70 (0.83–3.47)	0.145		
Clinical T stage				
T3–T4 vs. T2	2.30 (1.08–4.90)	0.031	2.38 (1.01–5.56)	0.046
Clinical N stage				
N1 vs. N0	0.67 (0.31–1.46)	0.314		
N2 vs. N0	0.86 (0.32–2.29)	0.756		
Clinical stage				
Ⅲ vs. Ⅰ–Ⅱ	0.72 (0.35–1.50)	0.378		
Distance from anal verge, cm				
5–10 vs. <5	1.84 (0.75–4.55)	0.184		
≥ 10 vs. <5	0.85 (0.36–2.05)	0.724		
Baseline neutrophil count (per 0.1 × 10⁹/L increase)	1.01(0.99–1.03)	0.191		
Baseline lymphocyte count (per 0.1 × 10⁹/L increase)	0.88 (0.83–0.93)	< 0.001	0.87 (0.81–0.93)	< 0.001
NLR ≥ 3	3.03 (1.38–6.68)	0.006		
BMI (per 1 kg/m^2^ increase)	0.93 (0.84–1.03)	0.149		
CEA > 5 ng/mL	1.49 (0.74–3.02)	0.267		
Concurrent chemotherapy^†^				
None vs. 5-FU/Capecitabine	0.32 (0.06–1.77)	0.190		
UFT vs. 5-FU/Capecitabine	0.63 (0.27–1.47)	0.289		
FOLFOX vs. 5-FU/Capecitabine	0.68 (0.28–1.62)	0.381		
RT dose > 50.4 Gy	0.59 (0.21–1.65)	0.313		
Lower pelvis dosimetry^†^				
V5 (per 1% increase)	1.05 (1.01–1.09)	0.020	1.06 (1.02–1.11)	0.005
V10 (per 1% increase)	1.03 (1.01–1.06)	0.019		

^†^ LP V10 was evaluated in univariable analysis but not included in multivariable analysis due to high collinearity with LP V5 and lower discriminative performance on ROC analysis

Abbreviations: ASL = acute severe lymphopenia; BMI = body mass index; CEA = carcinoembryonic antigen; CI = confidence interval; ECOG-PS = Eastern Cooperative Oncology Group performance status; FOLFOX = Oxaliplatin plus 5-fluorouracil/leucovorin; MVA = multivariable analysis; NLR = neutrophil-to-lymphocyte ratio; UFT = tegafur-uracil; UVA = univariable analysis; OR = odds ratio; Vx = volume of a structure receiving more than x Gy; RT = radiotherapy

**Fig. 2:**
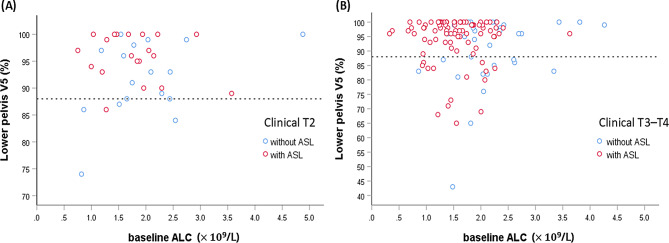
Distribution of patients with ASL according to baseline ALC and LP V5 values, stratified by clinical T stage: (**A**) T2 and (**B**) T3–T4. The dashed horizontal line denotes the optimal LP V5 cut-off of 88%. Patients who developed ASL tended to cluster above the V5 threshold and predominantly in the region of lower baseline ALC, indicating that both lower pretreatment lymphocyte levels and higher LP low-dose exposure contribute to ASL susceptibility. Compared with T2 cases, patients with T3–T4 disease showed a denser aggregation above this threshold, suggesting an overall higher propensity for ASL in the setting of more advanced local disease. Abbreviations: ALC = absolute lymphocyte count; ASL = acute severe lymphopenia; LP = lower pelvis; V5 = volume of LP receiving more than 5 Gy

To complement the continuous-variable analysis, a secondary model using the ROC-derived binary LP V5 cut-off (> 88% vs. ≤88%) was performed. Consistently, LP V5 > 88% remained significantly associated with ASL (OR 5.42, 95% CI 2.22–13.26; *p* < 0.001). Figure 2 illustrates the distribution of patients with ASL according to the baseline ALC and LP V5 values.

### The association of T stage with lymphocyte dynamics

Patients with a higher clinical T stage exhibited an increased risk of ASL. Although the baseline ALC appeared to decrease with increasing T stage (T2: 1.92 ± 0.81 × 10⁹/L; T3–T4: 1.69 ± 0.65 × 10⁹/L), this difference was not statistically significant (*p* = 0.076). Because the T stage was identified as an independent predictor of ASL after adjusting for baseline ALC and bone marrow dosimetry in multivariable regression, the greater lymphocyte depletion observed following radiation in a higher T stage disease is likely attributable to this association. To further explore the association between the ASL and T stage, we analyzed the CTVs for pelvic field and boost volumes in patients with IMRT. Patients with locally advanced disease (T3–T4) had significantly larger CTVs than those with T2 disease (CTV_pelvis_: 682.5 ± 170.8 c.c. vs. 628.2 ± 110.7 c.c., *p* = 0.024; CTV_boost_: 397.2 ± 138.4 c.c. vs. 343.0 ± 121.2 c.c., *p* = 0.033). However, both CTVs did not reach statistical significance in the logistic regression model. To further assess whether CTV volume could account for differences in low-dose marrow exposure, we evaluated the correlation between CTVs and LP V5 using Spearman’s coefficient. Only very weak, non-significant correlations were observed (CTV_pelvis_: ρ = 0.159, *p* = 0.051; CTV_boost_: ρ = 0.155, *p* = 0.058), indicating that variations in LP V5 are not solely explained by target volume.

## Discussion

This study demonstrates that ASL is independently associated with increased distant metastasis risk in rectal cancer following pelvic radiotherapy. These findings corroborate prior evidence on the prognostic significance of radiation-induced lymphopenia. Notably, ASL emerges as a modifiable toxicity linked to distant progression, with implications for radiotherapy planning. To our knowledge, this is the first study to specifically assess the impact of pelvic dosimetric parameters on ASL development and its association with survival outcomes in rectal cancer. This dosimetric focus represents a novel, actionable target for immune preservation in pelvic radiotherapy.

Accumulating evidence provides a biologically grounded explanation for the association between ASL and distant metastasis. Lymphocytes—particularly CD8⁺ cytotoxic and memory T cells—play essential roles in immune surveillance and cytotoxic effector function [22]. Radiation-induced lymphopenia compromises these functions through both direct cytotoxic depletion of circulating lymphocytes and irradiation-induced injury to lymphopoietic organs, including the pelvis-rich bone marrow and regional lymphatic structures. Preclinical models further demonstrate that T-cell depletion can drive metastatic outgrowth by shifting the tumor–immune equilibrium toward immune escape [23, 24]. Clinically, severe treatment-related lymphopenia has been linked to increased distant failure across multiple solid tumors [12–17], supporting the concept that systemic immune impairment facilitates metastatic progression. In this context, our finding that ASL predicts distant metastasis is consistent with these biological mechanisms and prior oncologic observations, suggesting a plausible mechanistic link between pelvic marrow irradiation, lymphocyte depletion, and metastatic dissemination. However, ASL may also reflect underlying disease aggressiveness and treatment intensity, and therefore the association observed in this study should be interpreted as correlation rather than proof of causality.

Low-dose irradiation to the LP bone marrow, particularly V5, emerged as a key determinant of ASL development in our study. Alongside baseline ALC—the strongest individual predictor—these findings underscore the radiosensitivity of lymphocytes and the immunological importance of dose exposure to lymphopoietic tissues. Prior studies in rectal cancer have primarily focused on high-dose PBM exposure (e.g., V40–45) and its association with hematologic toxicity, but these analyses largely emphasized non-lymphocyte lineages such as neutrophils or platelets [25–27]. Given that the pelvic bones contain approximately 40% of adult bone marrow [28], the broad nodal coverage required for pelvic malignancies often leads to substantial incidental irradiation. Even low-dose exposure can significantly impact lymphocyte integrity. Our findings support the need for lymphocyte-specific dose constraints, especially to anatomically distinct subsites like the LP marrow. In this context, the LymphoTEC guidelines have highlighted the importance of sparing low-dose regions (V5–V20) in lymphoid and blood flow-rich organs [29]. Supporting this, a cervical cancer study reported a 2.8-fold increase in ASL risk when PBM V10 exceeded 80% [30]. In our cohort, LP V5 was independently associated with ASL risk. The ROC-derived V5 threshold (≤ 88%) also demonstrated statistical significance in secondary binary analyses, reflecting dose levels associated with higher ASL risk. These results support incorporating LP-specific constraints into routine planning to preserve immune function. LP V5 captures the biologically relevant low-dose range most associated with lymphocyte radiosensitivity, which may explain its slightly superior discriminative performance compared with LP V10. Radiobiologic evidence demonstrates that circulating lymphocytes undergo profound depletion at dose exposures below approximately 5–10 Gy [31], supporting the clinical relevance of V5 as a practical and mechanistically grounded immune-sparing planning metric.

Beyond bone marrow dosimetry, disease burden may also influence the risk of ASL. In our study, higher clinical T stage was independently associated with ASL, suggesting a potential volume-driven effect related to tumor extent. Patients with T3–T4 disease exhibited significantly larger CTVs, which may increase radiation exposure to immune-relevant structures. These include not only pelvic marrow but also lymphatic and vascular structures, both of which are rich in circulating and tissue-resident lymphocytes. This “field-size effect” echoes findings in brain [12, 13] and thoracic cancers [14, 15, 17], where extended radiation volumes have been linked to greater lymphocyte depletion. However, correlation analyses demonstrated only very weak and non-significant associations between CTV volume and LP V5, suggesting that differences in CTV size alone do not fully account for the independent effect of T stage on ASL. While CTV metrics were not independently predictive in multivariable modeling, they likely interact with marrow dose-volume exposure and baseline immune status.

Because deaths without documented metastasis were censored in the Kaplan–Meier estimation, the incidence of distant failure may have been modestly underestimated. To assess methodological robustness, we additionally applied a Fine–Gray competing-risk model, which yielded results consistent with the Cox regression and confirmed the robustness of the ASL–DMFS association.

Although ASL was associated with worse DMFS, its impact on OS was limited. This discrepancy may be attributed to several factors. First, as OS events typically occur later than distant metastases, the current follow-up may be insufficient to capture late mortality differences, as suggested by the late crossover in KM curves (Fig. 1B). Second, effective systemic therapies—particularly adjuvant chemotherapy, which improved both DMFS and OS in our cohort—may have attenuated the detrimental effects of ASL by targeting micrometastases. In addition, post-metastasis salvage treatments may have further reduced survival differences [32]. Lastly, age, ECOG-PS and BMI—key OS predictors in our cohort—along with comorbidities, may have outweighed the influence of ASL on survival. To empirically evaluate whether this discordance reflected effect modification rather than absence of association, we conducted additional subgroup and interaction analyses. ASL was not associated with OS in either patients who did not receive adjuvant chemotherapy or those who received it, and interaction tests showed no significant modification of the ASL–OS relationship by age or ECOG-PS. A significant ASL–BMI interaction was observed, suggesting that higher BMI attenuated the adverse effect of ASL on OS. These findings indicate that the limited OS impact of ASL may be influenced by heterogeneous host factors rather than a uniform prognostic effect.

ASL incidence among patients with rectal cancer receiving CCRT typically ranges from 55% to 75%, in both neoadjuvant and adjuvant settings [27, 33, 34], aligning with our observed rate of 69.6%. This variability likely reflects differences in radiotherapy delivery techniques, particularly in low-dose bath beyond the target volumes. Rotational modalities such as VMAT and TOMO, which were used in nearly all patients at our institution, are favored for better OARs sparing but tend to generate larger low-dose volumes compared to static IMRT or 3DCRT [35, 36]. Without specific PBM constraints, this results in greater incidental irradiation to the LP marrow. Notably, TOMO plans were associated with higher LP V5 and lower V50 than VMAT, consistent with the technique’s low-dose bath effect [36]. These findings underscore the influence of technique on marrow dosimetry and support the integration of bone marrow–sparing strategies. The PRORECT trial further demonstrated that proton therapy significantly reduces low-dose exposure (V5–15 Gy) in short-course regimen for rectal cancer [37]. While these findings support the conceptual potential of proton therapy to mitigate low-dose bath, their applicability to conventional long-course chemoradiation remains uncertain. Accordingly, proton therapy should be viewed as a promising marrow-sparing strategy that warrants further evaluation in long-course settings, rather than as evidence for routine incorporation into clinical practice.

Our study uniquely quantifies how low-dose irradiation to the LP bone marrow contributes to ASL risk, providing a basis for targeted planning interventions. While clinical T stage and baseline ALC are intrinsic patient factors, radiation exposure to the LP marrow represents a modifiable parameter. These findings underscore the value of applying as low as reasonably achievable (ALARA) principles to this immunologically sensitive region. Notably, only continuous LP V5 remained independently predictive in the final multivariable model, establishing it as the primary modifiable dosimetric factor relevant to lymphocyte preservation. The ROC-derived V5 cut-off, although statistically significant in secondary analyses, should be regarded solely as an exploratory indicator of dose ranges associated with increased ASL risk rather than a definitive clinical threshold. Its appropriate use is within an ALARA framework—to guide dose minimization and inform planning goals—rather than to prescribe rigid constraints. Accordingly, this exploratory value may serve as an achievable planning target for high-risk patients but requires external validation before broader application.

This study has several limitations. First, its retrospective, single-center design may introduce selection bias, although inclusion of consecutive cases mitigates this risk. Second, the absence of an external validation cohort limits generalizability, and our findings require confirmation in prospective, multi-institutional settings. Third, since all patients received neoadjuvant or definitive treatment, applicability to adjuvant contexts remains unclear. Fourth, the near-universal use of rotational techniques precluded comparative analysis across modalities. Fifth, incomplete dosimetric data limited full-cohort analysis, though baseline characteristics were comparable between original and evaluable patients. Sixth, detailed metrics of chemotherapy exposure—such as relative dose intensity—were not available; although the distribution of concurrent chemotherapy regimens did not differ significantly between patients with and without ASL and chemotherapy regimen was not associated with ASL in univariable analysis, residual confounding from unmeasured chemotherapy-related myelotoxicity cannot be fully excluded. Seventh, immune profiling was restricted to basic lymphocyte/neutrophil counts and NLR, without assessing functional subsets such as T-cell phenotypes. Finally, the predictive performance of LP V5 was modest (AUC ~ 0.6) with limited specificity. The exploratory LP V5 cut-off requires external validation, as its performance may vary across institutions and planning techniques. Further refinement and integration with other clinical factors may improve their discriminatory capacity.

## Conclusions

Radiotherapy-related ASL independently predicts distant metastasis in rectal cancer. Key determinants include higher LP V5, lower baseline ALC, and advanced T stage. These findings highlight the importance of minimizing low-dose exposure to the LP marrow during treatment planning. Incorporating practical constraints based on ALARA principles may help mitigate this immunosuppressive effect, especially in high-risk patients. While our results support incorporating immune-sparing strategies, the modest predictive performance warrants further validation. Future studies should refine technical approaches and clarify the immunologic mechanisms linking ASL to disease progression.

## Supplementary Information

Below is the link to the electronic supplementary material.

Supplementary Material 1

## Data Availability

The datasets generated and/or analyzed during the current study are not publicly available due to Institutional Review Board restrictions, but are available from the corresponding author upon reasonable request in anonymized form, subject to data privacy review.

## Abbreviations

CCRT: Chemoradiotherapy
ASL: Acute severe lymphopenia
CBC: Complete blood count
OARs: Organs at risk
3DCRT: Three-dimensional conformal radiotherapy
IMRT: Intensity-modulated radiation therapy
VMAT: Volumetric modulated arc therapy
TOMO: Helical tomotherapy
CTV: Clinical target volume
PTV: Planning target volume
NLR: Neutrophil-to-lymphocyte ratio
PBM: Pelvic bone marrow
LS: Lumbosacral spine
ICs: Iliac crests
LP: Lower pelvis
Vx: Volume percentages receiving more than x Gy
DMFS: Distant metastasis-free survival
OS: Overall survival
ROC: Receiver operating characteristic
AUC: Area under the ROC curve
VIF: Variance inflation factor
UFT: Tegafur–uracil
5-FU: 5-fluorouracil
FOLFOX: Oxaliplatin plus 5-fluorouracil/leucovorin
ECOG-PS: Eastern Cooperative Oncology Group performance status
ALC: Absolute lymphocyte count
CEA: Carcinoembryonic antigen
CI: Confidence interval
HR: Hazard ratio
BMI: Body mass index
ALARA: As low as reasonably achievable
