# Supplementary material for: Radiation-induced severe lymphopenia predicts distant metastasis in rectal cancer: dosimetric implications for immune-sparing radiotherapy
Source: Radiat Oncol. 2026 Jan 29;21:34. doi: 10.1186/s13014-026-02791-3 (PMC12922384; doi:10.1186/s13014-026-02791-3)
Supplement: Supplementary file 1 — Supplementary Material 1 [file 13014_2026_2791_MOESM1_ESM.pdf]

Supplementary Material

1. Supplementary Figures

Figure S1 Flow diagram of study design and data collection. Abbreviation: RT = radiotherapy.

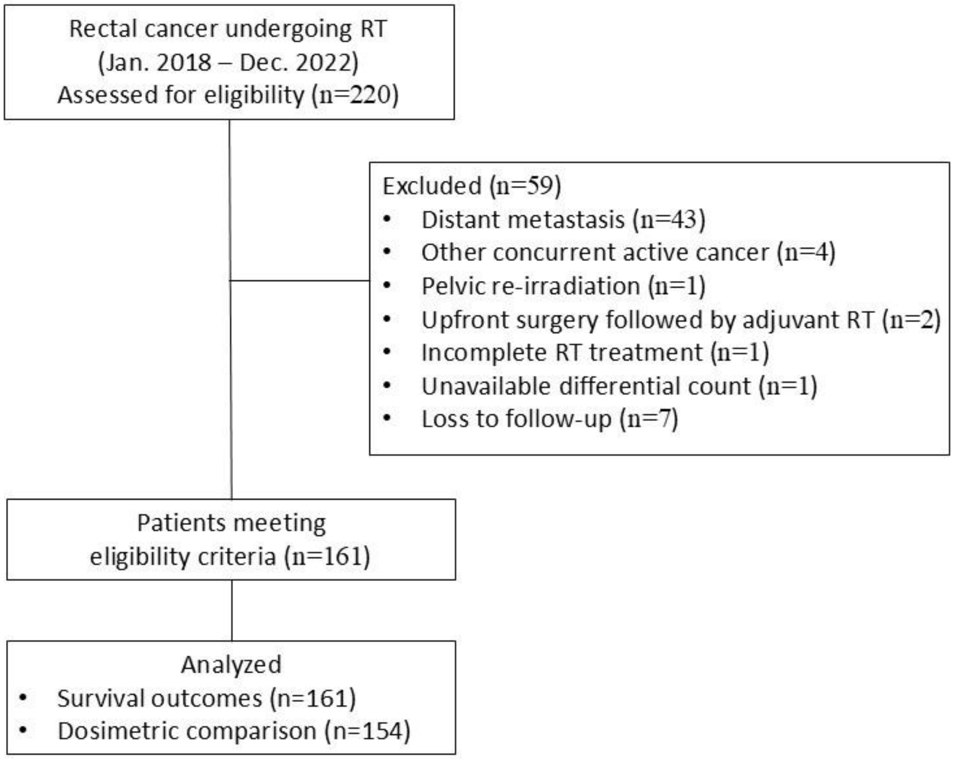

Figure S2 Cumulative incidence function curves for distant metastasis in patients with and without ASL, accounting for death as a competing risk. Patients with ASL showed a higher cumulative incidence of distant metastasis over time. Abbreviation: ASL = acute severe lymphopenia.

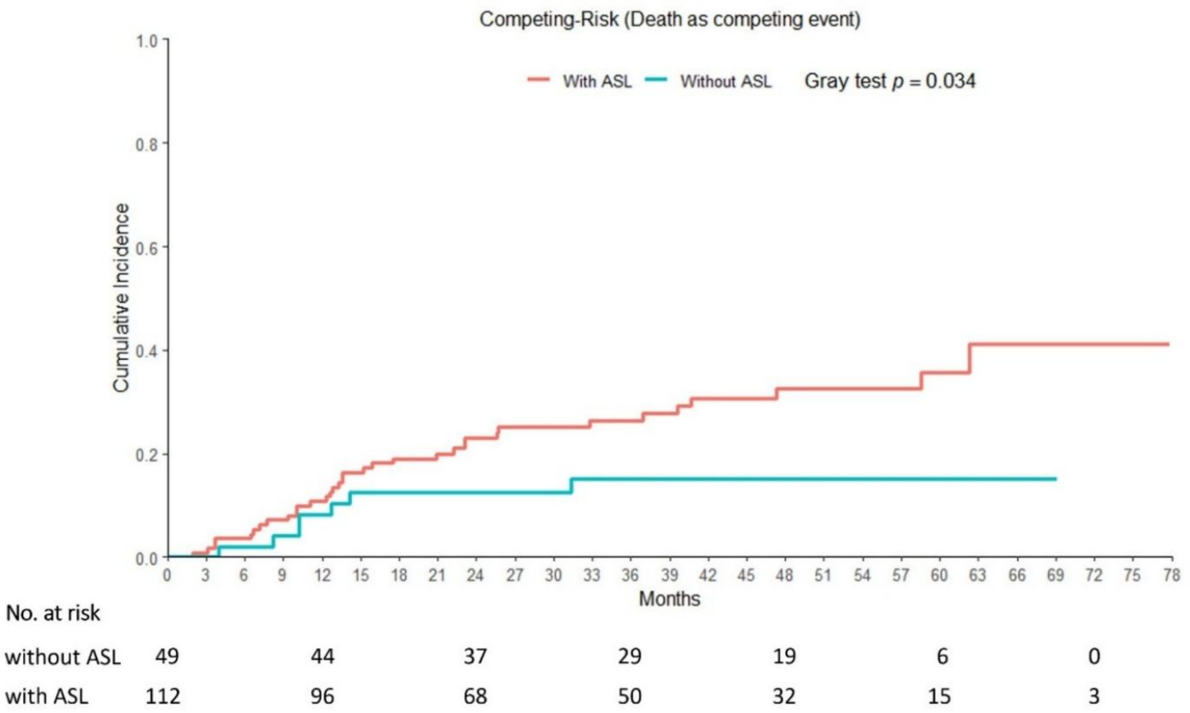

2. Supplementary Tables

Table S1 Comparison of cohort characteristics between patients with and without ASL

Parameter	Cohort without ASL (n = 49)	Cohort with ASL (n = 112)	*p*
Age >65 years	22 (44.9)	58 (51.8)	0.421
Male	36 (73.5)	69 (61.6)	0.146
ECOG-PS			0.175
0	34 (69.4)	64 (57.1)	
1	15 (30.6)	39 (34.8)	
2	0 (0)	7 (6.3)	
3	0 (0)	2 (1.8)	
Clinical T stage			0.029
2	17 (34.7)	21 (18.8)	
3	31 (63.3)	79 (70.5)	
4	1 (2.0)	12 (10.7)	
Clinical N stage			0.589
0	14 (28.6)	40 (35.7)	
1	26 (53.1)	50 (44.6)	
2	9 (18.4)	22 (19.6)	
Clinical stage			0.365
I	7 (14.3)	13 (11.6)	
II	7 (14.3)	27 (24.1)	
III	35 (71.4)	72 (64.3)	
Distance from anal verge, cm			0.133
<5	12 (24.5)	23 (20.5)	
5–10	15 (30.6)	53 (47.3)	
≥10	22 (44.9)	36 (32.1)	
Baseline blood cell count			
Leukocyte count, $\times 10^9$/L	7.24 ± 2.19	7.13 ± 2.33	0.769
Neutrophil count, $\times 10^9$/L	4.41 ± 1.79	4.88 ± 2.20	0.190
Lymphocyte count, $\times 10^9$/L	2.13 ± 0.80	1.58 ± 0.57	<0.001
NLR ≥3	10 (20.4)	49 (43.8)	0.005
BMI, kg/m^2	24.5 ± 3.5	23.7 ± 3.3	0.160
CEA >5 ng/mL	16 (32.7)	47 (42.0)	0.265
Concurrent chemotherapy			0.441
None	3 (6.1)	3 (2.7)	
5-FU	11 (22.4)	39 (34.8)	
Capecitabine	2 (4.1)	2 (1.8)	
UFT	18 (36.7)	36 (32.1)	
FOLFOX	15 (30.6)	32 (28.6)	
RT dose >50.4 Gy	7 (14.3)	10 (8.9)	0.309
RT fraction	28.2 ± 1.4	28.1 ± 1.3	0.595
RT duration, day	42.1 ± 7.5	41.7 ± 5.4	0.675

Data are presented as no. (%) or mean ± SD.

Abbreviations: ASL = acute severe lymphopenia; BMI = body mass index; CEA = carcinoembryonic antigen; ECOG-PS = Eastern Cooperative Oncology Group performance status; FOLFOX = Oxaliplatin plus 5-fluorouracil/leucovorin; NLR = neutrophil-to-lymphocyte ratio; RT = radiotherapy; SD = standard deviation; UFT = tegafur-uracil.

Table S2 Characteristics of original cohort and those evaluable for dosimetry

Parameter	Original cohort (n = 161)	Evaluable cohort (n = 154)	*p*
Age >65 years	80 (49.7)	75 (48.7)	0.861
Male	105 (65.2)	101 (65.6)	0.945
ECOG-PS			0.958
0	98 (60.9)	95 (61.7)	
1	54 (33.5)	51 (33.1)	
2	7 (4.3)	7 (4.5)	
3	2 (1.2)	1 (0.6)	
Clinical T stage			0.952
2	38 (23.6)	37 (24.0)	
3	110 (68.3)	106 (68.8)	
4	13 (8.1)	11 (7.1)	
Clinical N stage			0.988
0	54 (33.5)	51 (33.1)	
1	76 (47.2)	74 (48.1)	
2	31 (19.3)	29 (18.8)	
Clinical stage			0.997
I	20 (12.4)	19 (12.3)	
II	34 (21.1)	32 (20.8)	
III	107 (66.5)	103 (66.9)	
Distance from anal verge, cm			0.992
<5	35 (21.7)	34 (22.1)	
5–10	68 (42.2)	64 (41.6)	
≥10	58 (36.0)	56 (49.1)	
Baseline blood cell count			
Leukocyte, × 10^9/L	7.16 ± 2.28	7.22 ± 2.30	0.835
Neutrophil, × 10^9/L	4.74 ± 2.09	4.78 ± 2.10	0.869
Lymphocyte, × 10^9/L	1.75 ± 0.70	1.76 ± 0.69	0.877
NLR ≥3	59 (36.6)	56 (36.4)	0.959
BMI, kg/m^2	23.94 ± 3.36	23.95 ± 3.41	0.987
CEA >5 ng/mL	63 (39.1)	60 (39.0)	0.975
Concurrent chemotherapy			1.000
None	6 (3.7)	6 (3.9)	
5-FU	50 (31.1)	46 (29.9)	
Capecitabine	4 (2.5)	4 (2.6)	
UFT	54 (33.5)	52 (33.8)	
FOLFOX	47 (29.2)	46 (29.9)	
RT dose >50.4 Gy	17 (10.6)	16 (10.4)	0.961
RT fraction	28.2 ± 1.3	28.2 ± 1.3	0.925
RT duration, day	41.8 ± 6.1	41.7 ± 6.1	0.922
RT technique			0.999
3DCRT	2 (1.2)	2 (1.3)	
Static IMRT	4 (2.5)	4 (2.6)	
VMAT	135 (83.9)	128 (83.1)	

TOMO	20 (12.4)	20 (13.0)	
Surgery	120 (74.5)	115 (74.7)	0.977

Data are presented as no. (%) or mean ± SD.

Abbreviations: 3DCRT = 3D conformal radiotherapy; BMI = body mass index; CEA = carcinoembryonic antigen; ECOG-PS = Eastern Cooperative Oncology Group performance status; FOLFOX = Oxaliplatin plus 5-fluorouracil/leucovorin; IMRT = intensity-modulated radiotherapy; NLR = neutrophil-to-lymphocyte ratio; RT = radiotherapy; SD = standard deviation; VMAT = volumetric modulated arc therapy; TOMO = Helical Tomotherapy; UFT = tegafur-uracil.

Table S3 Comparison of dose volume parameters between VMAT and TOMO techniques

Dose volume parameter	VMAT (n = 128)	TOMO (n = 20)	*p*
Iliac crests			
V5, %	99.0 (95.0–100.0)	97.5 (93.5–100.0)	0.926
V10, %	92.0 (87.0–97.0)	96.0 (89.5–99.8)	0.055
V15, %	83.0 (77.0–88.8)	85.0 (78.3–92.8)	0.443
V20, %	71.0 (66.0–76.8)	71.0 (63.3–76.5)	0.768
V30, %	44.0 (36.0–50.0)	40.5 (28.5–44.0)	0.094
V40, %	21.0 (16.0–26.0)	19.5 (11.8–22.8)	0.167
V50, %	3.0 (1.0–5.8)	1.5 (1.0–5.0)	0.406
Dmean, Gy	27.7 (26.2–30.4)	27.9 (24.5–30.2)	0.519
Lumbosacral spine			
V5, %	99.5 (94.0–100.0)	98.5 (90.0–100.0)	0.262
V10, %	96.0 (86.0–100.0)	93.0 (82.8–99.8)	0.367
V15, %	93.5 (81.3–99.0)	91.0 (80.5–97.5)	0.441
V20, %	89.0 (77.3–97.0)	88.0 (76.3–95.3)	0.736
V30, %	77.5 (69.0–85.8)	80.0 (71.3–86.8)	0.741
V40, %	60.0 (53.0–68.0)	62.5 (51.3–74.5)	0.619
V50, %	23.0 (18.0–31.0)	26.0 (16.0–37.5)	0.728
Dmean, Gy	38.9 (35.4–42.0)	39.0 (35.8–42.3)	0.888
Lower pelvis			
V5, %	96.0 (89.0–99.0)	99.0 (97.0–100.0)	**0.007**
V10, %	87.0 (77.0–93.8)	91.0 (83.3–97.0)	0.088
V15, %	74.0 (61.3–84.0)	70.5 (62.8–74.0)	0.228
V20, %	57.0 (45.3–68.0)	53.5 (47.0–57.8)	0.147
V30, %	29.0 (21.3–35.0)	24.0 (19.3–28.8)	0.076
V40, %	11.0 (8.0–15.0)	10.0 (7.3–12.5)	0.228
V50, %	1.0 (0.0–2.0)	0.0 (0.0–1.0)	**0.030**
Dmean, Gy	23.7 (20.2–26.2)	22.6 (21.0–24.4)	0.335
Pelvic bone marrow			
V5, %	96.0 (92.0–99.0)	97.5 (93.5–99.8)	0.268
V10, %	90.0 (84.0–94.0)	91.0 (86.0–96.3)	0.237
V15, %	80.0 (74.0–87.0)	78.0 (72.8–84.8)	0.513
V20, %	68.5 (62.3–76.0)	66.0 (63.3–70.8)	0.308
V30, %	45.5 (40.0–52.0)	43.0 (36.0–50.3)	0.170
V40, %	26.0 (22.0–31.0)	26.5 (20.0–31.8)	0.570
V50, %	7.0 (5.0–10.0)	6.5 (4.3–11.5)	0.864
Dmean, Gy	28.6 (26.3–31.2)	27.9 (25.8–31.0)	0.606

Data are presented as median (25th–75th).

Abbreviations: ASL = acute severe lymphopenia; Vx = volume of a structure receiving more than x Gy; VMAT = volumetric modulated arc therapy; TOMO = Helical Tomotherapy
